# Therapeutic applications and characteristics of *Falcaria vulgaris* in traditional medicine and experimental studies

**DOI:** 10.22038/AJP.2021.18488

**Published:** 2022

**Authors:** Fatemeh Khazaei, Maryam Yadegari, Elham Ghanbari, Mohammadbagher Heydari, Mozafar Khazaei

**Affiliations:** 1 *Student Research Committee, Kermanshah University of Medical Sciences, Kermanshah, Iran*; 2 *Department of Anatomical Sciences, School of Medicine, Yazd University of Medical Sciences, Yazd, Iran*; 3 *Fertility and Infertility Research Center, Health Technology Institute, Kermanshah University of Medical Sciences, Kermanshah, Iran*; 4 *Department of Surgery, School of Medicine, Kermanshah University of Medical Sciences, Kermanshah, Iran*

**Keywords:** Falcaria vulgaris, Traditional medicine, Antioxidant

## Abstract

**Objective:**

*Falcaria vulgaris* is a herb with various applications in traditional medicine, including treatment of skin and gastric ulcers, liver diseases and gastrointestinal problems. It contains many valuable and important compounds with antioxidants and anti-ulcer properties, including carvacrol, spathulenol, limonene, tannins and saponins. In recent years, besides confirming many of its conventional uses, new beneficial properties of this plant have been identified. The purpose of this review is to investigate the therapeutic applications and botanical characteristics of *F. vulgaris* in traditional medicine and experimental studies.

**Materials and Methods:**

This study was a systematic review using the keywords "*Falcaria vulgaris*," "Therapeutic properties" and "Animal studies", 100 articles were extracted from various databases, including PubMed, SinceDirect, SID (scientific information database) and google search engines without time limit; after several stages of title monitoring and abstracts review, finally, 70 articles were selected for this study.

**Results:**

In traditional medicine of different countries, several therapeutic properties have been reported for *F. vulgaris*, most of which are attributed to its antioxidant content and the presence of tannins and saponins. In recent decades, many studies have been done to identify and confirm the medicinal properties of *F. vulgaris*, including antioxidant, antimicrobial and anti-diabetic effects, healing properties of skin and stomach ulcers, and protection of the liver and kidney.

**Conclusion:**

*F. vulgaris* has a variety of biological properties and is used as a valuable plant in medical research that helps to improve health and prevent some diseases.

## Introduction

Traditional medicine is one of the oldest methods of treatment in human societies and has been used by different nations long before modern medicine; it includes a set of skills and practices based on theories, beliefs and experiences of indigenous cultures. Traditional medicine is used in maintaining health, preventing and or treating various diseases (Kumari and Kotecha, 2016 ▶). The purpose of this science is to pay more attention to the plant and natural samples and their new applications as an adjuvant in the chemical treatment of diseases, to realize the therapeutic value of plants and finally to discover novel compounds such as antimicrobial, antiviral, antitumor and hormone-like compounds in plants. Furthermore, cultivation of medicinal plants is currently considered an important branch of agricultural economics that leads to extraction of beneficial compounds from plants and their use in treatment of diseases (Elansary et al., 2018 ▶).

Although chemical drugs are used to treat many diseases, their side effects, and incomplete ability to prevent and control diseases properly, as well as reduction of their effectiveness over time, have led researchers to explore new methods and discover appropriate ways to control diseases and their complications. In the United States, 2-3.6 million people use complementary and alternative medicine methods to treat the disease (Zhang et al., 2019 ▶) and among these different methods, the most attention is paid to herbal and dietary therapies. In this regard, Iranian traditional medicine has a history of several hundred years, which has received serious attention today (Henson et al., 2017 ▶).

**Figure 1:**
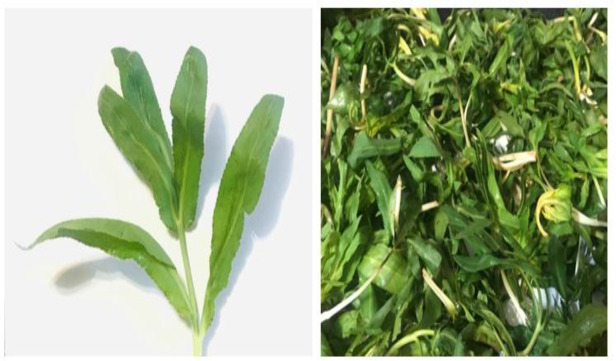
*Falcaria vulgaris* plant, photos were taken by author (F. Khazaei).

*Falcaria vulgaris*, also known as Ghazyaghi in Turkish and Paghazeh in the west of Iran, is a fast-growing plant of the Apiaceae (Umbelliferae) family with an average height of 30 cm (Behrooznia, 2019 ▶) (Figure 1). *F. vulgaris* grows as a weed in agricultural lands and is used as a spring vegetable in the diet in some parts of Iran, including the western provinces. In traditional medicine, various properties such as healing stomach and skin ulcers, and improving gastrointestinal and liver diseases, and kidney and bladder stones have been reported for the plant (Goorani et al., 2019b ▶). 

Considering the increasing value and special place of medicinal plants in pharmaceutical industries and the approach of communities towards the use of these plants and their derivatives (Kumar et al., 2013 ▶), the purpose of this review was to investigate therapeutic, pharmacological and phytochemicals properties of *F. vulgaris* plant in the treatment of different diseases in traditional medicine and experimental studies.

## Materials

This study was a systematic review with keywords "*Falcaria vulgaris*," "Therapeutic properties" and "Animal studies". One hundred articles from various databases, including PubMed, SinceDirect, SID (scientific information database) and Google search engine were extracted without a time limit. English and Persian articles about the role of the *F. vulgaris* extract in different diseases that had similar results or were not in line with the purpose of this study, including those reporting cultivation and maintenance methods of this plant, were excluded. 

**Figure 2:**
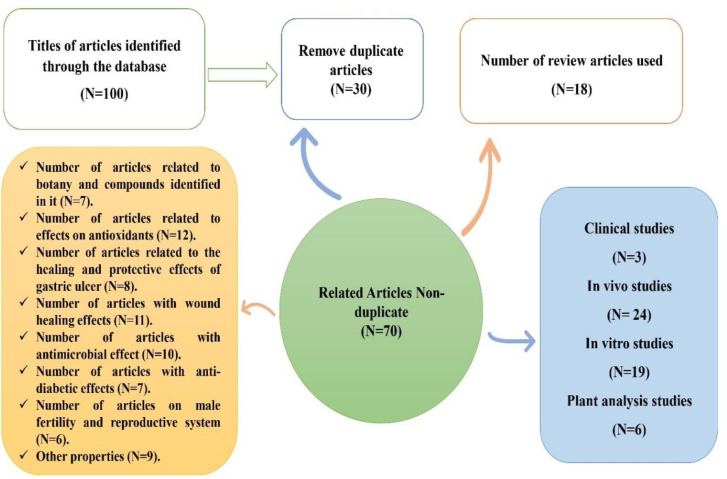
Flowchart of article information entry steps

After several stages of monitoring the titles, reviewing the abstracts and eliminating duplicates, according to Figure 2, seventy related articles were selected in this review study. These studies reported pharmacological properties of the plant, antioxidant, and anti-inflammatory, anti-cancer, and anti-bacterial activities, healing of skin and stomach ulcers, as well as its effect on the nervous and cardiovascular systems, diabetes, and liver, kidney, testicular and sperm parameters.

**Botanical characters**

*F. vulgaris* is a carnivorous, biennial herbaceous plant with a 20-30 cm tall hairless branched stem. The dark-green leaves resemble goose legs (Figure 1) and the lateral parts at the ends and edges are divided into linear spear pieces. It has a spindle-shaped and swollen root. Its flowers are white and pink, and its edible organ is the leaves (Amiri and Joharchi, 2016 ▶).

**Chemical compounds**

*F. vulgaris* contains highly volatile essential oils and a variety of proteins, starches and resins. The essential oils contain substances such as asparagines and a small amount of alkaloids and a large amount of sugar. Its resin contains phytosterols, phytosterolins, a mixture of fatty acids, including palmitic, stearic, and oleic and linoleic acids, and vitamin C (Shafaghat, 2010 ▶).

**Figure 3:**
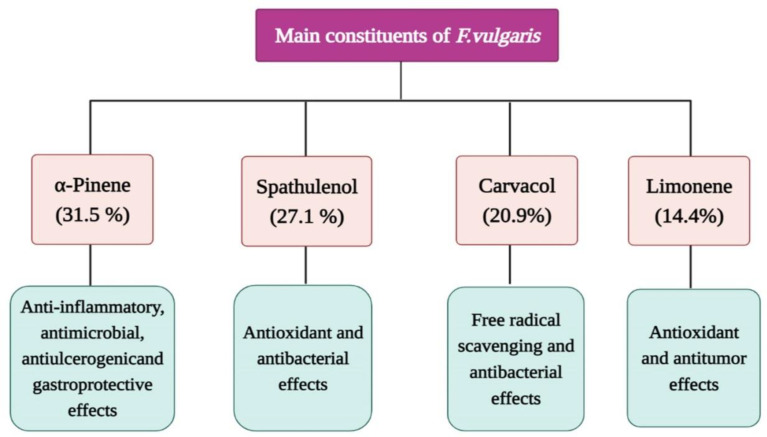
The main chemical constituents (%) of the *F. vulgaris* oil and their pharmacological effects

 A phytochemical study also showed the presence of tannins, saponins and monoterpenes in this plant (Khan Ahmadi and Shahrezaei, 2007 ▶). It was shown that α-pinene (31.5%), spathulenol (27.1%), carvacrol (20.9%) and limonene (14.4%) were the main constituents of the *F. vulgaris* leaf oil (Figure 3) (Asadbeigi et al., 2014 ▶).

**Antioxidant properties**

Free radicals have a highly reactive and unstable unpaired electron and can react rapidly with other molecules (Alkadi, 2020 ▶). They can cause tissue damage by reacting with unsaturated fatty acids in cell membranes and by reacting with DNA and cell proteins. They also cause oxidative stress by destroying the cellular structure (Zangeneh et al., 2018a ▶). Antioxidants reduce oxidative damage by scavenging free radicals or counteracting their damaging effects. These compounds may be synthesized in the body or supplied through a proper diet (Chang et al., 2016 ▶).

On the other hand, plasma antioxidant molecules come from two sources: endogenous (such as uric acid, albumin, and thiols) and exogenous, such as vitamins E and C. Total antioxidant capacity (TAC) shows the total activity of antioxidant groups in plasma and body fluids (Pisoschi and Negulescu, 2011 ▶). TAC can protect cells against free radical damage and may be used to prevent and cure a variety of diseases (Gonçalves et al., 2019 ▶).

The most common active ingredients in herbs are phenolic compounds, vitamins, terpenoids (carotenoids and triterpenes) and alkaloids. Some of these compounds have strong antioxidant activity (Amiri and Joharchi, 2016). Phenolic antioxidants react with free radicals faster than biomolecules (proteins, DNA, and membranes) to inhibit them to protect cells from oxidative stress and prevent the formation of reactive oxygen/nitrogen species (Ivanišová et al., 2020 ▶). 

*F. vulgaris* has antioxidant effects and can protect cells from oxidative damage by neutralizing reactive oxygen species (ROS). The antioxidant activity of this plant is attributed to its phenolic compounds (Jaberian et al., 2013 ▶). Investigation of the antioxidant power of oils extracted from the stem and flower of *Falcaria* showed that these compounds eliminate free radicals, and due to the high content of monoterpenes, they have higher antioxidant activity compared to other oils (Shafaghat, 2010 ▶).

*F. vulgaris* extract (FVE) is a considerable source of active phenolic compounds that have high antioxidant capacity and free radical scavenging power. Furthermore, its phenolic compounds prevent lipoprotein oxidation (Monfared et al., 2012 ▶). In a study, a high-fat diet decreased the activity of antioxidant enzymes and increased ROS in rat liver tissue. Treatment with FVE significantly increased the activity of antioxidant enzymes catalase, superoxide dismutase and glutathione peroxidase (Goorani et al., 2019c ▶). 

Studies have shown that FVE is rich in spathulenol, carvacrol, alpha-pinene and limonene as strong anti-inflammatory and antioxidant components. It contains water-insoluble phenolic monoterpenes, such as carvacrol and alpha-pinene, and has a wide range of medicinal properties for example antioxidant, antibacterial, anti-viral, anti-inflammatory, anti-depressant and immune-boosting properties. These compounds are also used in food and production of medicines (Jaberian et al., 2013 ▶). 

Toxic chemical compounds and some drugs have adverse effects on the body's metabolism. Carbon tetrachloride (CCl_4_) can cause liver damage. FVE reduces the production of free radicals and clears them. It increases cellular antioxidant stores and may play an important role in protecting liver cells from harmful *in vivo* agents. FVE (160 mg/kg for 45 days) reduced hepatic oxidative stress and increased the activity of antioxidant enzymes (Zangeneh et al., 2018b ▶). 

Another study showed that the aqueous-alcoholic extract of *F. vulgaris* leaves can be used to produce copper nanoparticles. Metal nanoparticles have strong antioxidant activities and eliminate all types of free radicals. It was also shown that FVE alone and its green synthesis of copper nanoparticles had the activity of inhibiting free radicals similar to the synthetic antioxidant butylated hydroxytoluene (BHT) (Zangeneh et al., 2019 ▶) (Table 1).

Since free radicals interact with the cell membrane by the peroxidation of unsaturated fatty acids induced pathological changes, *F. vulgaris* may play an important role in their protections. The results of studies showed that the bioactive compounds of the *F. vulgaris* prevent the activity of free radicals caused by metabolic diseases and/or aging, and reduce oxidative stress. Therefore, this medicinal plant can be considered a natural source to improve the side effects caused by diseases.

**The protective and healing effect of the plant on gastric ulcer**

Nowadays, gastrointestinal diseases are one of the most common disorders in different countries. Gastric/peptic ulcer is a multifactorial disease with benign mucosa and submucosa lesions. The risk of peptic ulcer increases by steroid factors, psychological stress, smoking, use of non-steroidal analgesics and anti-inflammatory drugs, *Helicobacter pylori* infection, and alcohol and caffeine consumption (Bhoumik et al., 2017 ▶). 

Treatment of gastric ulcers with various chemical drugs is expensive and has different side effects; also, it is often associated with the return of lesions after cessation of treatment. For this reason, the use of medicinal plants is of special importance, and herbal compounds have provided promising results for gastric ulcers treatment (Wang et al., 2019 ▶). 

In several experimental animal studies, we investigated the protective and healing effect of FVE against gastric ulcers. First, the protective effect of FVE (50, 100 and 150 mg/kg) on ethanol (50%)-induced gastric ulcer was established (Khazaei and Salehi, 2006 ▶). Then, its protective effect (150 mg/kg) against aspirin-induced (200 mg/kg) gastric lesions was confirmed (Khazaei et al., 2006 ▶). In another study, the healing effect of FVE on aspirin-induced gastric ulcer was investigated, and the extract significantly reduced the wound index, improved the structural changes of gastric tissue and increased collagen fiber formation compared to aspirin and omeprazole groups (Yadegari et al., 2015 ▶). These studies demonstrated that *F. vulgaris* helps in healing gastric ulcers.

**Table 1:** Biological and pharmacological effects of *F. vulgaris*

Authors (Year)	Type of extract	Parts of *F. vulgaris* (Bioactive compounds)	Extract dosage	Finding
	Hydro-alcoholic	Aerial parts	50, 100, 150 mg/kg	Protective effect against gastric ulcer
	Hydro-alcoholic	Aerial parts	150 mg/kg	Healing of aspirin induced gastric ulcer
	Hydro-alcoholic	Tannin	5% and 10%	Wound healing
Khan-Ahmadi and Shahrezaei (2007) ▶	Oil	Aerial parts	----	Carvacrol (20.93%) Spatulenul (27.08%)
	Hydro- ethanolic	Leaf	0.2, 1 and 5 µg/ml	Coronary artery dilatation
	Oils extracted	Aerial parts	----	Free radicals' scavenger
	Oil	Spatolinol and carvacrol	0.1% -0.2%	Antibacterial activity
	Hydro-alcoholic	Aerial parts	150 mg/kg	Antifertility effect in female rat
	Hydro- ethanolic	Phenolic compounds	-----	Antioxidant, free radicals' scavenger
	Hydro-methanolic	Phenolic compounds and carvacrol	-----	Antioxidant
	Hydro-alcoholic	Aerial parts	150 mg/kg	Microscopic finding on aspirin-induced gastric ulcer
	Ethanolic	Plant essential oil	50, 70, 80% methanol and ethanol	Antimicrobial effect and strong antioxidant activity
	Aqueous	Leaf	10%	Skin wound healing
	Aqueous	Aerial parts	40, 80, 160, 320, 640 and 1280 µg/ml	Anti-leishmaniasis
	Hydro- ethanolic	Aerial parts	25-100 mg/ml	Antibacterial (Gram-negative and positive)
	Aqueous	Aerial parts	200, 600 and 1800 μg/kg	Antidiabetic, nephro-protective and hematoprotective
	Hydro- ethanolic	Leaf	20, 50, 100 mg/kg	Increase prolactin and milk production
	Aqueous	Leaf aqueous FVE (nanoparticles)	2-8 mg/ml	Free radical scavenging activity and antioxidants
	Aqueous	FVE (nanoparticles)	4-8 mg/ml	Antifungal and bacterial
	Aqueous	Leaf	25, 50, 100 and 200 mg/kg	Anti-anemic
	Aqueous	Leaf	1-1000 μg/ml	Cutaneous wound treatment
	Aqueous	Leaf	25, 50, 100 and 200-mg/kg	Treat fatty liver
	Aqueous	aqueous FVE (nanoparticles )	0.535 µg/ml	Antibacterial (MDR bacteria)
	Aqueous	Titanium nanoparticles	216 mg/ml	Antioxidant, antifungal, antibacterial, and cutaneous wound healing

**Skin wound healing effect**

The skin is the largest organ in the human body and plays important roles in protection, waste disposal, and vitamin D synthesis. Loss of part of the skin can cause secondary lesions and diseases (Naghsh et al., 2013 ▶). Thus, one of the most important issues that medical science has faced so far is problems of wound healing. Increasing the healing rate of wounds has multiple economic and health benefits; plants and their derivatives have long been used to treat and manage various types of wounds (Jivad et al., 2016 ▶). 

The process of skin wound healing is a dynamic, complex and regular reaction that includes platelet aggregation, blood coagulation, angiogenesis, inflammatory response to injury and regeneration of epithelial tissue (Guo and DiPietro, 2010 ▶). Inflammation is a common phenomenon in the wound healing process and is very important for killing infectious microorganisms such as bacteria and fungi. When germs clearance is incomplete, bacteria and endotoxins can increase inflammatory cytokines and prolong the inflammatory phase of repair. Therefore, it is important to identify drugs that accelerate regeneration of the epidermis and dermis against skin damage (Yaghoobi and Kazerouni., 2013 ▶). 

The use of *F. vulgaris* in the treatment of skin wounds dates back to several decades ago. Recent research has reported significant antiseptic activity of FVE without side effects, and its efficacy in treatment of skin lesions caused by leishmaniasis (Eskandarian et al., 2017 ▶). However, more studies are needed. Another study showed that green synthesis copper nanoparticle from aqueous FVE is very effective in killing fungal and bacterial pathogens and healing skin wounds (Zangeneh et al., 2019 ▶). *F. vulgaris* aqueous extract ointment also significantly reduced the size of the wound and significantly increased wound contracture (Goorani et al., 2019b ▶).

Limonene is a monoterpene with antitumor, antibiotic and antimicrobial activity and has anti-leishmania effects (Arruda et al., 2009 ▶). A previous study showed alpha-pinene, and limonene has anti-leishmania activities (Soares et al., 2013 ▶). *F. vulgaris* is rich in these compounds. In addition, saponin is another compound of this plant with anti-leishmaniasis effects (Britta et al., 2014 ▶). 

F. vulgaris has a substantial influence on the healing process of skin wounds by modifying the immune system and may has beneficial effect in skin wounds caused by bacterial and viral diseases (Choobkar, 2015 ▶).

*F. vulgaris *helps to speed up the healing of skin wounds by raising total antioxidant capacity, and improving blood flow to the wound area (Choobkar, 2015 ▶). In other studies, FVE accelerated the healing of skin wounds and peptic ulcers, with increasing proliferation of fibroblasts and further collagen synthesis (Tahvilian et al., 2007 ▶). The results of a study showed that FVE is effective in accelerating wound healing (Shakibaei et al., 2007 ▶) (Table 1).

F. vulgaris has a sufficient anti-leishmaniasis effect and can effectively heal wounds without causing side effects. As a result, it can be used as a supplement or replacement for cutaneous leishmaniasis. However, more *in vivo* research is needed. Furthermore, the excellent antimicrobial ability of synthesis gold nanoparticles from FVE enhanced cutaneous wound healing while reducing the proliferation of bacteria and fungi in the wound (Zangeneh et al., 2019a ▶).

**Antimicrobial effects**

Some medicinal plants have antimicrobial properties and numerous therapeutic capabilities in the treatment of infectious diseases, and are one of the valuable resources in medicine. As a result of the spread of these diseases, identifying such plants and purifying their effective compounds can be helpful (Mazzei et al., 2020 ▶). It decreases a huge number of negative effects that are typically connected with the use of antibiotics in the treatment of infectious diseases and other disorders (Semeniuc et al., 2017 ▶). 

Minimum bactericidal concentration (MBC) and Minimum fungicidal concentration (MFC) are the lowest concentrations, which inhibit bacterial or fungal growth respectively. Synthesis copper nanoparticles from aqueous FVE suppressed the activity of fungi (*C. krusei, C. glabrata* and *C. albicans*) at concentrations of 2–4 mg/ml, and inhibited the growth of bacteria (*S. typhimurium, E. coli, S. aureus, P. aeruginosa, S. pneumoniae *and *B. subtilis*) at 2–8 mg/ml concentrations, indicating that they have antibacterial properties (Zangeneh et al., 2019 ▶).

Research has shown that the main constituents of *F. vulgaris*, spathulenol and carvacrol are part of sesquiterpene and monoterpenes, respectively, and have antimicrobial activity (Choobkar et al., 2017 ▶). Evaluation of the effect of FVE on fungi and bacteria of *Candida albicans*, *C. glabrata, C. krusei, C. guilliermondii, Pseudomonas aeruginosa, Escherichia coli, Staphylococcus aureus, *and *Bacillus subtilis*, has shown that its antifungal and antibacterial effects are more than the antibiotics tested (Zangeneh et al., 2019 ▶).

 Furthermore, the plant essential oils consist of volatile phenolic compounds and antioxidants that have strong antimicrobial properties. *F. vulgaris* essential oil has antibacterial properties and can be used as an inexpensive source to treat some bacterial infections. It can also be considered a suitable alternative to synthetic antibiotics. However, more studies are needed to study the antibacterial effect of *F. vulgaris* and its compounds more closely (Shafaghat, 2010 ▶).

Bazzaz et al. also showed that *F. vulgaris* has antibacterial effects against *Escherichia coli*, *Klebsiella pneumoniae*, *Salmonella typhi*, *Pseudomonas aeruginosa*, *Morganella morganii*, *Bacillus subtilis*, *Staphylococcus aureus*, and *Candida albicans* (Bazzaz and Haririzadeh, 2003 ▶). In another study, the antimicrobial effect of FVE was investigated; Based on the disk diffusion method results, growth inhibitory effect of ethyl acetate extract of *F. vulgaris* was higher than other extracts of this plant. In bioautography of the extract, the antimicrobial effect on Gram-positive bacteria (*Staphylococcus aureus* and *Staphylococcus epidermidis*) was higher than gram-negative bacteria (*Klebsiella pneumoniae *and* P. aeruginosa*). 

The minimal inhibitory concentration (MIC) of the total extract was obtained for *S. aureus*, *Salmonella typhi* and *Bacillus licheniformis* 8 mg/ml. MIC was 16,000 μg/ml for *Pseudomonas aeruginosa* and *Bamilus subtilis* and 32,000 μg/ml for *Escherichia coli* and *Klebsiella pneumonia* (Moshafi et al., 2015 ▶).* F. vulgaris* has good antimicrobial activity against many pathogenic bacteria. The diameter of the growth inhibition zone of antibiotics was the highest of different concentrations of aqueous and ethanolic FVE in the Kirby-Bauer disk diffusion method. The results of the least concentration of growth inhibitor and minimum concentration of lethality demonstrated that the ethanolic FVE has a more inhibitory and lethal impact than aqueous extract at lower concentrations. This effect can be attributed to the fact that more effective antimicrobial compounds are obtained in ethanolic extract (Pourhaji and Nyasti, 2017 ▶).

Research has shown that the antibacterial properties of many of these plants are mainly related to the phenolic compounds in the extracts and essential oils, including thymol and carvacrol (Zhang et al., 2018 ▶). Carvacrol, like other phenolic compounds, has an antibacterial effect lead to bacterial death due to membrane damage (Adel et al., 2016 ▶). *F. vulgaris* is one of the medicinal plants that has a high percentage of carvacrol (29.8%) and can be a source of natural products with antibacterial and antioxidant activity (Shanmuganathan et al., 2018 ▶).

Nosocomial infections have caused many problems in terms of the failure of treatment and mortality in patients due to antibiotic resistance (Darvishi et al., 2020 ▶). Hekmati et al. investigated the antibacterial effect of *F. vulgaris*, *Allium rotundum* and *Ferulago angulate* *Boiss* extracts on the *P. aeruginosa *and* S. aureus* causing nosocomial infections, and reported that the studied extracts had a strong antimicrobial effect. 

The amount of MIC in FVE decreased, and produced silver nanoparticles similarly reduced the MIC level in plant extract, indicating that this compound increased antibacterial activity (Hekmati et al., 2020 ▶). 

The phenols, long chains of terpenes and alcohols such as oleic acid, spathulenol, methoxsalen, carvacrolin the extract of that have antimicrobial effects and can be used as adjunctive therapy in infections caused by multidrug resistant (MDR), extensively drug resistant (XDR) and pandrug resistant (PDR) (Hekmati et al., 2020 ▶).

Another study showed that the aqueous extract of *F. vulgaris* can be used in the easy and green preparation of silver nanoparticles (AgNP). They reported that synthesis AgNPs from *F. vulgaris* had the lowest antibacterial activity against *E. coli* (MDR) and the highest activity in inhibiting *S. aureus*. Therefore, *Staphylococcus aureu*s ATCC 25923 bacteria except for *P. aeruginosa *(MDR) were more sensitive to AgNP-Fv than multiple drug-resistant bacteria. These NPs can be used in various medical aspects (Ahmeda et al., 2020 ▶; Kohsari et al., 2019 ▶).

Synthesis titanium nanoparticles from the aqueous FVE showed excellent antioxidant, antibacterial, antifungal and cutaneous wound healing properties. MBC and MIC of these nanoparticles against bacteria were at 2-16 mg/ml and 2-8 mg/ml, respectively. It had the highest antifungal and antibacterial effects on *C. krusei* and *B. subtilis,* respectively. Nanoparticles at 2, 4, and 8 mg/ml concentrations inhibit *C. guilliermondii*, *B. subtilis*, *S. aureus*, *C. krusei, S. pneumoniae, C. albicans, C. glabrata, E. coli*, *P. aeruginosa *and* S. typhimurium* growth, and destroyed *C. guilliermondii/ B. subtilis/C. krusei, C. glabrata/S. pneumoniae, C. albicans/S. aureus*, and *E. coli/ S. typhimurium/P. aeruginosa *at 2, 4, 8, and 16 mg/ml concentrations, respectively (Ahmeda et al., 2020 ▶).

A pervious study showed that fungi and bacteria tested, including C*. glabrata, S. typhimurium, P. aeruginosa, S. aureus, *and* C. albicans*, are sensitive to synthesis silver nanoparticles from aqueous FVE and these nanoparticles showed more antibacterial and antifungal than reference antibiotics (Tetracycline, Ceftriaxone, Penicillin, Gentamycin, Cefpirome). Therefore, these nanoparticles due to their non-toxicity and antioxidant, antifungal, antibacterial and skin wound healing effects can be well used for therapeutic and industrial purposes (Hekmati et al., 2020 ▶). As a result, these studies reveal that FVE has antioxidant, antibacterial, and antifungal properties. This plant extract contains bioactive compounds that are known to be free radical scavengers and inhibit Gram-positive, Gram-negative, and fungal strains.

**Anti-diabetic effects**

Diabetes is a chronic disease that causes high blood sugar and metabolic disorders due to a lack or decrease in insulin function. Epidemiological observations have shown that the prevalence of diabetes has increased with changes in dietary culture and lifestyle from traditional to industrial life (Makalani et al., 2017 ▶). In many cases, proper diet and alternative therapies prevent the advancement of type 2 diabetes in addition to reducing treatment costs (Adedapo and Ogunmiluyi, 2020 ▶).

Currently, there are several drugs to lower blood glucose, but due to the lack of a complete cure for this disease, the tendency to use alternative and traditional therapies has increased, and the role of herbs with hypoglycemic properties in the treatment of diabetics cannot be ignored. The use of herbs by diabetic patient is widespread even in Western countries, especially when conventional therapies are not able to control the disease, and the patient needs to receive insulin (Khazaei et al., 2018).

High blood glucose and oxidative stress are two diabetic side effects that are linked to one another (Chen et al., 2011 ▶). Saponin, found in many natural plants and compounds, can treat diabetes due to its antioxidant and antiglycemic properties. Also, tannin has antioxidant effects and inhibits lipid peroxidation (Ravi et al., 2017 ▶). Research suggests that diabetes-induced anemia increases glycosylation of the red blood cell (RBC) plasma membrane. On the other hand, protein oxidation causes increased lipid peroxidation of RBC membranes, which leads to increased membrane hardness and decreased fluidity and flexibility, resulting in hemolysis of RBCs. Treatment of diabetic rats with FVE (200, 600 and 1800 μg/kg), especially at a dose of 200 μg/kg, reduced blood glucose and improved changes in blood parameters (mean corpuscular hemoglobin, mean corpuscular hemoglobin concentration, mean corpuscular volume and red blood cells) due to diabetes (Zangeneh et al., 2018a ▶).

Oxidative stress is strongly associated with diabetes. *F. vulgaris* contains antioxidants, and its extract, by inhibiting the production of ROS, can reduce malondialdehyde levels and increase total antioxidant capacity. Therefore, due to the presence of saponin and other antioxidant compounds, *F. vulgaris* can have positive effects in terms of reducing diabetes-induced oxidative stress (Salahshoor et al., 2019 ▶). As a result, determining the active components in this plant and administering appropriate doses can help in the treatment of diabetes.

**Protective effects on the liver and kidney**

The liver and kidneys are two important organs that are continually active in detoxifying and eliminate the harmful effects of medicines and their metabolites. As a result, it is critical to identify compounds that protect these organs from dangerous chemical compounds and drug side effects*. F. vulgaris* is one of the most effective natural compounds for protecting these organs from harmful agents. Administration of carbon tetrachloride (1 mg/kg) changed biochemical parameters, including liver function enzymes such as alanine aminotransferase (ALT), alkaline phosphatase (ALP) and aspartate aminotransferase (AST), platelet and white blood cells count, cholesterol, and low and high-density lipoprotein in mice. The use of different doses of *F. vulgaris*, especially 160 mg/kg, improved the harmful effects of carbon tetrachloride on the mentioned parameters and showed liver protection effects (Zangeneh et al., 2018b ▶). 

Increased free radicals in liver cells caused inflammatory response and infiltration of mononuclear inflammatory cells into damaged liver tissue. FVE reduced the activity of liver enzymes ALT, ALP and AST in rats, which indicates inhibition of liver damage caused by diabetes. The results of histopathological examination of liver tissue also showed that the lobular and cellular structure of the liver was normal in the diabetic group receiving *F. vulgaris* extract (Salahshoor et al., 2019 ▶). 

Furthermore, A high-fat diet causes severe hepatotoxicity and a significant increase in the degree of hepatic steatosis, an increase in cholesterol, LDL, triglycerides, ALP, AST, ALT, gamma-glutamyl transferase (GGT), and conjugated and glycosylated bilirubin, and a significant decrease in total protein and albumin compared with the control group. Treatment with aqueous FVE (200 mg/kg) significantly improved the above-altered parameters (Goorani et al., 2019c ▶). 

Acute kidney damage following STZ administration increased urea and creatinine concentrations in mice. However, administration of FVE could significantly reduce the amount of urea and creatinine (Zangeneh et al., 2018a ▶). Chronic alcohol use reduces renal tubular reabsorption and decreases renal function, which is confirmed by elevated plasma levels of creatinine and urea. Glomerular filtration rate (GFR) decreased after kidney injury. FVE use improved renal dysfunction induced by alcohol consumption, such that combining the extract with alcohol considerably lowered serum creatinine and urea concentrations, as well as improved alterations in renal tissue caused by alcohol consumption (Zangeneh et al., 2018a ▶).

Treatment of liver and renal disorders with available drugs may be associated with side effects and development of new protective drugs is essential. Therefore, due to its antioxidant properties, *F. vulgaris* can be used to produce new drugs for the prevention and treatment of liver or kidney diseases with the least side effects. Therefore, further research including clinical trials to evaluate the effects of this plant, is needed in this regard.

**Cardiovascular effect**

Numerous studies have proven the cardiovascular effects of herbs. *F. vulgaris* has been shown to reduce cardiac ischemia in rats, with doses of 7.5, 15, and 22.5 mg/kg and significantly increasing coronary fluid flow, and changes in left ventricular pressure, heart rate. Furthermore, a major change of ventricular diastolic pressure has not seen (Shakibaei and Goudini, 2007 ▶). In another study, FVE significantly increased coronary blood flow in isolated rat heart. However, the extract was injected into the isolated heart for a longer period of time and at a higher concentration, it can be regarded safe. Furthermore, the effect of FVE on coronary artery dilation can be emphasized (Shackebaei and Godini, 2009 ▶).

**Effect on fertility**

Infertility is one of the most important issues in a couple's life. Male infertility is caused by problems with the production, maturation, and motility of sperm (Ghanbari et al., 2020 ▶). Most herbs are rich in antioxidant compounds that increase motility and maintain sperm morphology. Antioxidants protect sperm from free-radical damage and improve sperm quality. STZ, as a diabetes inducer, can disrupt testicular histology and sperm parameters, *F. vulgaris* extract increased sperm viability, and improved their number, morphology, and motility in diabetic rats. Therefore, FVE could be useful for reducing the complications of diabetes in men and enhancing male fertility (Roshankhah and Jalili, 2018). 

*F. vulgaris* has positive effects on milk production, expression of prolactin receptor (PRLR) gene, and serum prolactin and breast tissue in favor of increased milk production in rats. However, the mechanism of this extract effect on milk production parameters is not clear (Salahshoor et al., 2018 ▶). Some phytoestrogens reduce the amount of progesterone. These compounds exert their direct effects by modulating the function of steroid-producing enzymes (Kim and Park, 2012 ▶). 

Hydroxysteroid dehydrogenase is a key enzyme in the production of progesterone (Gaskins and Chavarro, 2018 ▶). Flavonoids also inhibit the activity of this enzyme through the cAMP pathway and ultimately reduce the production of progesterone in the ovaries. Progesterone produced by the corpus luteum promotes uterine survival and protection during pregnancy, its decrease prevents the ability to preserve the fetus (Ożarowski et al., 2019 ▶). Yadegari et al. observed that injection of FVE (150 mg/kg) in pre-fertilization, pre-implantation and implantation periods, reduced the number of implantation sites and the number of neonates. The mechanism of *Falcaria *contraceptive and abortion effect is unknown. However, the contractile effect of tannin can be considered (Yadegari et al., 2011 ▶). 

**Other properties**

Anemia is one of the most common and widespread diseases in the world that has different types and causes, and to some extent, different treatments. Many herbs have been suggested for the prevention and treatment of anemia in traditional medicine due to the body's tolerance to natural medications and the lack of significant side effects. Goorani et al. reported anti- anemia effect of FVE which increases the red blood cell count, mean hemoglobin concentration and mean corpuscular volume (Goorani et al., 2019a ▶).

**Side effects**

Herbal remedies cannot be considered entirely safe since they are natural goods. Herbal medicines may create adverse effects such as headaches, nausea, vomiting, allergic reactions and diarrhea that can vary from mild to severe. *F. vulgaris*, like other medicinal herbs, has some side effects, particularly on the reproductive system. Yadegari et al. (2011) ▶ showed abortive effect of high levels of *F. vulgaris* extract during implantation periods (sensitive time of pregnancy). 

## Conclusion

*F. vulgaris* contains saponins, tannins, carvacrol and spathulenol, which have antioxidant properties and are effective in treating stomach and skin ulcers, diabetes, infections, and liver and kidney disorders. Due to its therapeutic benefits and numerous biological properties, and low cost compared to chemical drugs, its use in the diet of many patients can be considered by the treatment team. However, the use of extracts and compounds of this plant as a medicine for the prevention and treatment of diseases requires additional clinical trials. 

## Conflicts of interest

The authors have declared that there is no conflict of interest.
